# What Are the Predictors of Intracranial Aneurysm Rupture in Indonesian Population Based on Angiographic Findings? Insight from Intracranial Aneurysm Registry on Three Comprehensive Stroke Centres in Indonesia

**DOI:** 10.1155/2022/4787048

**Published:** 2022-03-17

**Authors:** Jovian P. Swatan, Achmad F. Sani, Dedy Kurniawan, Hermanto Swatan, Shakir Husain

**Affiliations:** ^1^Department of Neurology, Faculty of Medicine Universitas Airlangga, Surabaya, Indonesia; ^2^Department of Neurology, Dr. Soetomo General Hospital, Surabaya, Indonesia; ^3^Department of Neurology, Faculty of Medicine Widya Mandala Catholic University, Surabaya, Indonesia; ^4^Director of Stroke and Neurointervention Foundation (SNIF), New Delhi, India

## Abstract

**Objectives:**

What are the Predictors of Intracranial Aneurysm (IA) Rupture based on angiographic findings among patients in Indonesia's Population Based on Angiographic Findings.

**Materials and Methods:**

We conducted a cross-sectional study on subjects with IA not caused by congenital aetiologies or other vascular malformations with cerebral angiography performed from January 2017 to January 2021. Demographic data and aneurysm profile, which include aneurysm count, size, location, and rupture event, were collected. The correlation between risk factors and IA rupture events was determined using bivariate and multivariate analysis.

**Results:**

From 100 angiography data (33 males and 67 females), the mean subject age is 51.94 ± 10.78. We observe a total of 121 IAs from all subjects. Most of the IAs are in the anterior circulation (104 aneurysms, 85.96%), have small size (77 aneurysms, 63.64%), and are found in ruptured conditions (90 aneurysms, 74.38%). Males have a greater aneurysm count (1.36 ± 0.74 vs. 1.13 ± 0.55, *p* = 0.036) and larger aneurysm size (*p* = 0.002). Aneurysm size is significantly correlated with its location (*p* = 0.008). Medium size (*p* = 0.019; OR 2.62, 95% CI 1.08-6.36) and location other than the internal carotid artery are associated with increased rupture event. Multivariate analysis revealed that gender (*p* = 0.031; aOR 5.37, 95% CI 1.17-24.70) is a significant risk factor of IA rupture event.

**Conclusion:**

IA profiling will enable clinicians to determine the risk of rupture and treatment plans for the Indonesian population. Further studies with a larger sample size are required to confirm these findings.

## 1. Introduction

Intracranial aneurysm (IA) is the most common central nervous system vascular malformation with a prevalence of 0.4-3% among the general population. If left untreated, IA could evolve into subarachnoid haemorrhage (SAH) which poses significant mortality and morbidity [1]. According to several studies in Indonesia, ruptured aneurysmal SAH has a mortality rate of 20.8-53.1% [2–4], which is significantly higher compared to other Southeast Asian countries [5, 6]. Recently, the fast development of technological advances could significantly reduce the mortality and morbidity level of ruptured IA through early and aggressive treatment. Several treatment options to treat IA include endovascular coiling or surgical clipping [2].

The urgency of establishing an IA profile for the Indonesian population lies in the high morbidity and mortality caused. By the completion of this study, there is no published study with an adequate sample size regarding IA profile among the Indonesian population. Therefore, this study is aimed at analysing and stratifying IA rupture risk through cerebral angiography data to provide insight into the demographic conditions and IA profiles in Indonesia.

## 2. Materials and Methods

### 2.1. Study Design

This is the first IA profiling study in the Indonesian population, which is an extension of a previously published study [7]. All datasets obtained in this study are stored in the Indonesia National Aneurysm and Cerebral Vessel (INACV) Registry, which is an active registry continuously updated with IA and cerebral vessel profiles from comprehensive stroke centres around Indonesia.

A cross-sectional study was conducted on October–November 2021 in three comprehensive stroke centres in Surabaya, Indonesia. We analysed retrospectively all cerebral angiography data obtained from January 2017 up to January 2021. All subjects with IA were included in this study. Subjects with other vascular malformation, congenital malformation, extracranial aneurysms, and incomplete datasets were excluded. This study complies with the Strengthening the reporting of observational studies in epidemiology (STROBE) guidelines.

We collected demographic data and IA profiles in all subjects. Demographic data collected include gender, age, cerebral angiography modalities, diagnostic indication, and treatment received. Meanwhile, the IA profile collected includes aneurysm count, size, neck and dome ratio, location, and rupture event.

### 2.2. Data Management

We classified aneurysm size into 4 categories based on the largest dimension of IA in millimetres, namely, small (<5.00 mm), medium (5.00-14.99 mm), large (15.00-24.99 mm), and giant (25.00 mm and above) [8]. IA was categorized as a wide-neck aneurysm if the neck size ≥ 4 mm and/or dome-to-neck ratio < 2 [9]. IA rupture event was classified into ruptured and unruptured.

During bivariate and multivariate analysis, the data used were stratified as follows: age group was stratified into 2 (≤50 and >50); aneurysm location was categorized into 5, namely, internal carotid artery (ICA), anterior communicating artery (Acom), middle cerebral artery (MCA), posterior communicating artery (PCom), and other location.

### 2.3. Statistical Analysis

The acquired data were analysed using IBM SPSS Statistics for Windows ver. 23.0 (IBM Corp, Armonk, USA). Descriptive data were expressed as mean ± standard deviation. Aneurysm profile and demographic data were compared using *t*-test, Mann–Whitney *U*-test, Fisher exact test, and Kruskal-Wallis test. A *p* value of <0.05 was considered statistically significant.

Bivariate and multivariate analysis was conducted, respectively, using the chi-square test and logistic regression. We analysed gender, age group, aneurysm size, and aneurysm location in correlation with the rupture risk. Risk was presented as odds ratio (OR) and adjusted OR with 95% confidence interval (CI).

### 2.4. Ethical Clearance

This study followed the principles of the Declaration of Helsinki and had received ethical exemption from Dr. Soetomo General Hospital Health Research Ethics Committee (ethical clearance no. 0606/LOE/301.4.2/IX/2021) before it began. Details that might disclose the subject's identity were omitted.

## 3. Results

### 3.1. Demographic Data

We obtain a total of 114 cerebral angiography data within the time period. However, 14 data are excluded due to incomplete datasets. The mean age of 100 subjects (33 male and 67 female) is 51.94 ± 10.78. Digital subtraction angiography is the most common modality used for cerebral angiography, with subarachnoid haemorrhage as the most common indication. A total of 14 IAs are found incidentally in subjects with ischemic stroke (10 subjects), diplopia (1 subject), tinnitus (1 subject), epilepsy (1 subject), and medical check-up without any presenting symptoms (1 subject). All of the incidental IAs are in unruptured condition. We also found that most subjects have one aneurysm and receive medication with endovascular coiling. The demographic data are presented in Table 1.

### 3.2. Aneurysm Profile

We collect a total of 121 IAs among subjects. Most of the aneurysms found are small in size, have wide neck, and are located in the anterior circulation. A total of 90 ruptured IAs are found in 86 subjects, with 4 subjects having two ruptured IAs. The profile of IA among subjects is shown in Table 2.

### 3.3. Aneurysm Profile and Demographic Data

#### 3.3.1. Aneurysm Profile and Gender

We observe that males have a higher mean aneurysm count than females (1.36 ± 0.74 vs. 1.13 ± 0.55, mean rank 55.73 vs. 47.93, *p* = 0.036). Regarding the highest aneurysm size in a subject, we found that males have a significantly larger aneurysm size than females (mean rank 61.48 vs. 45.09, *p* = 0.002). However, the distribution of wide-neck aneurysm among genders is statistically insignificant (*p* = 0.211). The crosstabulation of gender and aneurysm profile is shown in Table 3.

#### 3.3.2. Aneurysm Profile and Age Group

We find no significant difference in mean age between male and female subjects (50.33 ± 11.26 vs. 52.73 ± 10.54, respectively, independent *t*-test *p* = 0.298). We classify the subjects into 5 age groups as shown in Table 1. There is no significant difference in aneurysm count and size among age groups (Kruskal-Wallis test, *p* = 0.266 and 0.911, respectively).

#### 3.3.3. Aneurysm Size and Location

We observe that small aneurysms are most common in Acom (28.57%) and ICA (23.38%) while medium aneurysms in PCom (35.71%) and Acom (16.67%). We also identify one large aneurysm in ICA and one giant aneurysm in VBJ. The relationship between aneurysm size and location is statistically significant (Kruskal-Wallis test, *p* = 0.008).

#### 3.3.4. Aneurysm Rupture Risk Analysis

Bivariate analysis of IA rupture risk is summarized in Table 4. We exclude large and giant aneurysms for analysis because of the small number. We observe a significant correlation of IA rupture risk with aneurysm size (*p* = 0.019; OR 2.62, 95% CI 1.08-6.36) and location other than ICA. However, there is no significant correlation between age group and gender with increased rupture risk.

We conduct multivariate analysis only for single aneurysms (Table 5). The variables included are gender, age group, aneurysm size, and location. We use backward likelihood ratio logistic regression to analyse the variables. We found that gender is significantly correlated with increased rupture risk (*p* = 0.031, aOR 5.37, 95% CI 1.17-24.70). The impact of this variable on the risk of rupture was 12.0%.

## 4. Discussion

SAH is widely known as the most devastating type of stroke with high mortality and morbidity. Previous studies suggested higher mortality rates of SAH in Indonesia [2–4] compared with studies in other Southeast Asian countries [5, 6] and worldwide [10]. Several factors that may contribute to this figure include (1) sociodemographic factors, namely, low awareness of health issues and hesitancy to conduct routine medical check-up if no symptoms are present [11–13]; (2) diagnostic and treatment delay due to the cultural belief to self-medicate and disparities in healthcare facility and personnel across this archipelago nation [14, 15]; and (3) study design and location, as our hospital is the tertiary health care and referral centres for eastern Indonesia region and most of the SAH patients usually admitted with severe disease progression and other complications that could not be handled in primary or secondary health centres. Although we do not analyse the SAH mortality rates, the high incidence of ruptured IA compared to previous studies [16, 17] may illustrate the magnitude of SAH burden in Indonesia.

Most of the subjects with IA in this study are female. This finding is similar with previous studies where females have significantly higher IA prevalence compared with males [18–20]. One hypothesis stated that hormonal factors might have a significant role in vessel wall remodelling after menopause [21, 22]. This theory also supports our findings where the mean female age in this study is above the median age of natural menopause [23]. This study is in line with previous studies where males are associated with larger aneurysm size [22, 24, 25] but contradicts those where females are associated with multiple IA count [25–27].

The mean age of subjects in this study is 51.94 years old with a wide distribution. This finding is similar with previous studies where the mean age of patients with IA ranged from 50 to 57 years old [27–29]. However, we observe no significant relationship between age group with IA count and size. These findings were supported by several studies [28, 30–32], but different from previous studies where elderly in the age group 70 and older had an increased risk of ruptured IA [29, 33].

In this study, we report a significant relationship between aneurysm location and size where PCom had the highest mean IA size compared with other locations. Previous studies also reported this significance of size and distribution; however, the highest mean IA size was reported in ICA and MCA [34, 35]. We argue that these differences may be attributed to different population characteristics and geographical locations of subjects in previous studies.

We decide to analyse gender, age group, aneurysm size, and location in relation to the rupture risk based on result from previous studies. Previous studies supported our findings where larger aneurysm and location other than ICA are attributed to increased rupture incidence [34–38]. Several studies suggested that measuring aneurysm size in relation to the parent vessels may be a better predictor of rupture incidence [39, 40].

Previous studies have reported that multiple IAs may contribute to increasing the risk of rupture [41, 42]. However, we exclude multiple IAs in multivariate analysis. This consideration was made because we analyse both demographic and aneurysm profiles simultaneously. Thus, we sought to provide a more reliable rupture risk analysis for each individual aneurysm by analysing only single aneurysms. When analysing the variables simultaneously, we found that gender is significantly correlated with increased rupture risk. Although this finding is in line with previous studies [43, 44], we found that the rupture risk is much higher in our study. Again, different population subgroup and location may be an argument to this finding.

This study has several limitations. Firstly, data reported in this study may not fully represent Indonesia's heterogeneous population. Although one of the comprehensive stroke centres in this study is a national referral centre for the eastern part of Indonesia, we still have no adequate data to stratify the risk based on each population group. Secondly, the composition of ruptured and unruptured IA in this study is uneven. This may be attributed to the lack of awareness in health screening and conducting routine cerebral angiography in a population when no symptoms are present. Thirdly, the limited demographic data and clinical profiles obtained in this study due to data analysis were based only on cerebral angiography results. Further studies are required to correlate patients' sociodemographic and clinical characteristics with IA profile.

## 5. Conclusions

IA profiling is an urgent and a matter of great importance for Indonesia's population. Analysis of IA data will provide insight to clinicians and researchers regarding the aneurysm risk stratification map among Indonesian populations. Further studies involving multiple comprehensive stroke centres throughout Indonesia in addition to subjects' clinical features are needed to accurately predict the aneurysm rupture risk and decide the best treatment for individual subjects.

## Figures and Tables

**Table 1:** Demographic data of subjects.

Demographic data (*n* = 100)	*N* (%)
Gender	
Male	33 (33.00)
Female	67 (67.00)
Age	
Mean	51.94 ± 10.78
Younger than 40	14 (14.00)
40–49	23 (23.00)
50–59	42 (42.00)
60–69	16 (16.00)
70 and older	5 (5.00)
Diagnostic modality	
Computed tomography angiography	3 (3.00)
Magnetic resonance angiography	3 (3.00)
Digital subtraction angiography	94 (94.00)
Diagnostic indication	
Subarachnoid haemorrhage	65 (65.00)
Intracerebral haemorrhage	9 (9.00)
Subarachnoid haemorrhage and intracerebral haemorrhage	12 (12.00)
Others	14 (14.00)
Aneurysm count	
1	86 (86.00)
2	10 (10.00)
3	2 (2.00)
4	1 (1.00)
5	1 (1.00)
Treatment	
Best medical treatment	46 (46.00)
Medication+endovascular coiling	51 (51.00)
Medication+surgical clipping	3 (3.00)

**Table 2:** Intracranial aneurysm profile.

Subject IA profile (*n* = 121)	*N* (%)
Size (mm)	
Neck	
Mean	3.08 ± 1.71
Range	0.60–9.30
Dome	
Mean	4.41 ± 3.22
Range	0.40–26.40
Length	
Mean	4.64 ± 3.41
Range	0.70–23.90
Size category	
Small	77 (63.64)
Medium	42 (34.70)
Large	1 (0.83)
Giant	1 (0.83)
Dome-to-neck ratio	
Mean	1.52 ± 0.86
Range	0.29–5.17
Wide-neck aneurysm	
Yes	98 (80.99)
No	19 (15.70)
Not applicable for measurement	4 (3.31)
Rupture incidence	
Ruptured	90 (74.38)
Unruptured	31 (25.62)
Location	
Anterior circulation	104 (85.96)
Internal carotid artery (ICA)	20 (16.53)
Anterior cerebral artery (ACA)	5 (4.13)
Anterior choroidal artery (AChoA)	6 (4.96)
Anterior communicating artery (Acom)	29 (23.97)
Middle cerebral artery (MCA)	19 (15.70)
Posterior communicating artery (PCom)	25 (20.66)
Posterior circulation	17 (14.04)
Posterior cerebral artery (PCA)	4 (3.31)
Basilar artery (BA)	5 (4.13)
Superior cerebellar artery (SCA)	2 (1.65)
Anterior inferior cerebellar artery (AICA)	1 (0.83)
Posterior inferior cerebellar artery (PICA)	1 (0.83)
Vertebrobasilar junction (VBJ)	2 (1.65)
Vertebral artery (VA)	2 (1.65)

**Table 3:** Crosstabulation of aneurysm profile and gender.

Aneurysm profile	Male (*n* = 33) *N* (%)	Female (*n* = 67) *N* (%)	*p* value
Aneurysm count			0.036^a^^∗^
1	25 (75.76)	61 (91.04)	
2	5 (15.15)	5 (7.47)	
3	2 (6.06)	0 (0.00)	
4	1 (3.03)	0 (0.00)	
5	0 (0.00)	1 (1.49)	
Largest aneurysm size			0.002^a^^∗^
Small	12 (36.36)	45 (67.16)	
Medium	19 (57.58)	22 (32.84)	
Large	1 (3.03)	0 (0.00)	
Giant	1 (3.03)	0 (0.00)	
Wide-neck aneurysm			0.211^b^
Yes	30 (90.91)	55 (82.09)	
No	2 (6.06)	11 (16.42)	
Not applicable for measurement	1 (3.03)	1 (1.49)	

^a^Using the Mann–Whitney *U*-test; ^b^using the Fisher exact test. ^∗^*p* < 0.05.

**Table 4:** Bivariate analysis of all intracranial aneurysm and rupture risk.

Parameter	Ruptured, *n* (%)	OR	
		*p*	95% CI
Gender			
Male	25/31 (80.65)	—	Ref
Female	63/67 (94.03)	0.068	3.24 (0.99–10.67)
Age			
≤50 years	35/39 (89.74)	—	Ref
>50 years	53/59 (89.83)	1.000	1.01 (0.30–3.34)
Aneurysm size			
Small	53/77 (68.83)	—	Ref
Medium	37/42 (88.10)	0.019^∗^	2.62 (1.08–6.36)
Aneurysm location			
ICA	9/21 (42.86)	—	Ref
Acom	25/29 (86.21)	0.004^∗^	7.64 (1.93–30.21)
MCA	15/19 (78.95)	0.035^∗^	4.58 (1.12–18.80)
PCom	19/25 (76.00)	0.037^∗^	3.87 (1.09–13.81)
Other location (AChoa, ACA, and posterior circulation)	22/26 (84.62)	0.007^∗^	6.72 (1.69–26.78)

^∗^Significant for *p* < 0.05.

**Table 5:** Multivariate analysis of single intracranial aneurysm and rupture risk.

Parameter	aOR			*R* square
	*B*	*p*	95% CI	
Gender	1.681	0.031	5.37 (1.17-24.70)	12.0%
Constant	1.281	0.011		

^∗^Significant for *p* < 0.05.

## Data Availability

The data used to support the findings of this study are available from the corresponding author upon request.

## Supplementary Materials

Supplementary Materials S1: completed STROBE checklist
