# Supplementary material for: What Are the Predictors of Intracranial Aneurysm Rupture in Indonesian Population Based on Angiographic Findings? Insight from Intracranial Aneurysm Registry on Three Comprehensive Stroke Centres in Indonesia
Source: Stroke Res Treat. 2022 Mar 17;2022:4787048. doi: 10.1155/2022/4787048 (PMC8947878; doi:10.1155/2022/4787048)
Supplement: Supplementary Materials — S1: completed STROBE checklist [file 4787048.f1.docx]

**Supplementary Materials**

**S1 –** STROBE Checklist

|  | Item No | Recommendation | Page, Line Number | Comment |
| --- | --- | --- | --- | --- |
| **Title and abstract** | 1 | (*a*) Indicate the study’s design with a commonly used term in the title or the abstract | Title of the Research (Page 1, line 2-5) | The title of research includes specific terms according to the keywords indexed by MeSH.  (Keywords: Cerebral Angiography, Indonesia, Intracranial Aneurysm, Risk Factors Analysis, Ruptured Aneurysm) |
|  |  | (*b*) Provide in the abstract an informative and balanced summary of what was done and what was found | Heading: Abstract  Subheadings: Method and Result  (Page 1, line 17-31) | An informative summary of this research has been described in the abstract. The abstract has given brief details about what was done during the study in **methods** section and all the findings in the **result** section |
| **Introduction** | | | | |
| Background/rationale | 2 | Explain the scientific background and rationale for the investigation being reported | Heading: Introduction  (Page 2, line 37 - 50) | The introduction part mentioned the magnitude of ruptured intracranial aneurysm among population and highlight the needs of IAs profiling due to lack of IA data in current population. |
| Objectives | 3 | State specific objectives, including any prespecified hypotheses | Heading: Introduction  (Page 2, line 48 - 50) | The aim of this study has been mentioned, which is to analyse and stratify IA rupture risk through cerebral angiography data to provide insight into the demographic conditions and IA profiles in Indonesia |
| **Methods** |  |  |  |  |
| Study design | 4 | Present key elements of study design early in the paper | Heading: Material and Methods  (Page 2, line 52-67) | The main study design was cross-sectional and the elements of the design was described in this section |
| Setting | 5 | Describe the setting, locations, and relevant dates, including periods of recruitment, exposure, follow-up, and data collection | Heading: Material and Methods  (Page 2, line 58-67) | - Setting of location: Research was conducted in 3 comprehensive stroke centres in Surabaya, Indonesia - Setting of time: All cerebral angiography data obtained from January 2017 – January 2021. - Recruitment period: N/A - Exposure: N/A - Follow-up: N/A - Data collection: Data collection was conducted on October – November 2021. |
| Participants | 6 | (*a*) Give the eligibility criteria, and the sources and methods of selection of participants | Heading: Material and Methods  (Page 2, line 58-62) | - Eligibility criteria: Cerebral Angiography data of all subjects with Intracranial aneurysms - Exclusion criteria: Other vascular malformation, congenital malformation, extracranial aneurysms, and incomplete dataset. - Sources: Cerebral Angiography database - Sampling methods: Total sampling |
| Variables | 7 | Clearly define all outcomes, exposures, predictors, potential confounders, and effect modifiers. Give diagnostic criteria, if applicable | Heading: Material and Methods  (Page 2, line 64-77) | - Outcome: Significance between aneurysm profile and demographic data. - Variable of interest: Demographic data (age, gender), IA Profile (including Count, Size, Location, and Rupture event) - Diagnostic criteria: Aneurysm size measured in mm and classified into 4, namely small (< 5.00mm), medium (5.00-14.99mm), large (15.00-24.99 mm), and giant (25.00mm and above); Wide-neck aneurysm if the neck size ≥ 4 mm and/or dome-to-neck ratio < 2 |
| Data sources/ measurement | 8* | For each variable of interest, give sources of data and details of methods of assessment (measurement). Describe comparability of assessment methods if there is more than one group | Heading: Material and Methods  (Page 2, line 68-77) | - Data sources: Cerebral Angiography Database - Measurements: Details regarding classification of aneurysm size, wide-neck, and rupture event had been elaborated in the paper. |
| Bias | 9 | Describe any efforts to address potential sources of bias | Heading: Material and Methods  (Page 2, line 58-59) | This study was conducted in 3 comprehensive stroke centres in Surabaya, Indonesia. |
| Study size | 10 | Explain how the study size was arrived at | Heading: Material and Methods  (Page 2, line 59-60) | Total sampling of all cerebral angiography data conducted from January 2017 – January 2021. |
| Quantitative variables | 11 | Explain how quantitative variables were handled in the analyses. If applicable, describe which groupings were chosen and why | Heading: Material and Methods  (Page 2, line 69-73) | Classification of IA size was based on Indonesia National Consensus of Interventional Neurology (2020)  Classification of Wide-neck aneurysm was based on Zhao et al (2016). |
| Statistical methods | 12 | (*a*) Describe all statistical methods, including those used to control for confounding | Heading: Material and Methods  (Page 3, line 78-86) | Aneurysm profile and demographic data were compared using t-test, Mann-Whitney U-test, Fisher Exact Test, and Kruskal Wallis Test.  Bivariate and multivariate analysis was conducted respectively using chi-square test and logistic regression. |
|  |  | (*b*) Describe any methods used to examine subgroups and interactions | X | N/A |
|  |  | (*c*) Explain how missing data were addressed | X | There were no missing data in this study |
|  |  | (*d*) If applicable, describe analytical methods taking account of sampling strategy | X | No follow-up study was conducted |
|  |  | (*e*) Describe any sensitivity analyses | X | N/A |
| **Results** | | | | |
| Participants | 13* | (a) Report numbers of individuals at each stage of study—eg numbers potentially eligible, examined for eligibility, confirmed eligible, included in the study, completing follow-up, and analysed | Heading: Results  (Page 3, line 94-95) | Potentially eligible: 114  Examined for eligibility: 114  Confirmed eligible: 100  Included in the study: 100  Completing follow-up: N/A  Analysed Data: 100 |
|  |  | (b) Give reasons for non-participation at each stage | Heading: Results  (Page 3, line 94-95) | 14 data were excluded due to incomplete dataset |
|  |  | (c) Consider use of a flow diagram | X | N/A |
| Descriptive data | 14* | (a) Give characteristics of study participants (eg demographic, clinical, social) and information on exposures and potential confounders | Heading: Results  (Page 3, line 103) | Demographic data of subjects has been described on Table 1 |
|  |  | (b) Indicate number of participants with missing data for each variable of interest | X | N/A |
| Outcome data | 15* | Report numbers of outcome events or summary measures | Heading: Results  (Page 3, line 93-141) | Analysis of Demographic data, IA profile, and rupture event have been described on the result section |
| Main results | 16 | (*a*) Give unadjusted estimates and, if applicable, confounder-adjusted estimates and their precision (eg, 95% confidence interval). Make clear which confounders were adjusted for and why they were included | X | N/A |
|  |  | (*b*) Report category boundaries when continuous variables were categorized | Heading: Results  (Page 4, line 103, 109, 135) | Subjects age were categorized into 5 subgroups,  Aneurysm Size was classified into 4 subgroups.  Aneurysm Location was further classified into two locations (Anterior and Posterior Circulation)  In bivariate and multivariate analysis, age was classified into 2 (≤50 and >50), Aneurysm Location was classified into 5 (ICA, Acom, MCA, Pcom, Other Location). |
|  |  | (*c*) If relevant, consider translating estimates of relative risk into absolute risk for a meaningful time period | X | N/A |
| Other analyses | 17 | Report other analyses done—eg analyses of subgroups and interactions, and sensitivity analyses | Heading: Material and Methods  (Page 3, line 78-86) | Analysis has been described on the methods section. |
| **Discussion** | | | | |
| Key results | 18 | Summarise key results with reference to study objectives | Heading: Discussion  (Page 6, line 142-194) | We have summarized key result and compared it with previous studies in the discussion section. |
| Limitations | 19 | Discuss limitations of the study, taking into account sources of potential bias or imprecision. Discuss both direction and magnitude of any potential bias | Heading: Discussion  (Page 7, line 186-194) | Limitations for this study include sample representation and limited sociodemographic data and clinical profile analysed. |
| Interpretation | 20 | Give a cautious overall interpretation of results considering objectives, limitations, multiplicity of analyses, results from similar studies, and other relevant evidence | Heading: Discussion  (Page 6, line 142-194) | - Objectives: Analyse and stratify IA rupture risk through cerebral angiography data to provide insight into the demographic conditions and IA profiles in Indonesia. - Limitations: study conducted only in 3 Comprehensive Stroke Centres which may not fully represent Indonesia population, limited sociodemographic data and clinical profile analysed. - Multiplicity of analyses: N/A - Results from similar studies: N/A - Other relevant evidence: similar and contradictory evidence from previous studies have been described in the discussion section |
| Generalisability | 21 | Discuss the generalisability (external validity) of the study results | Heading: Conclusions  (Page 8, line 195-201) | Analysis of IA data will provide insight to clinicians and researchers regarding the aneurysm risk stratification map among Indonesian populations. |
| **Other information** | | | | |
| Funding | 22 | Give the source of funding and the role of the funders for the present study and, if applicable, for the original study on which the present article is based | Heading: Funding Statement  (Page 8, line 207-209) | - Funding source: This study did not receive any funding or grants from specific organization and was not performed as a part of the employment of the authors |
